# Sister Tutors for Mental Hospitals

**Published:** 1921-09-24

**Authors:** 


*To the Editor of* The Hospital.

### Sister-Tutors for Mental Hospitals.

*To the Editor of* The Hospital.

Sir,—I read with much interest your article upon sister-tutors for mental hospitals, and should hope that there will be one stipulation made if such a post materialises—that she hold the M.P.S. certificate. (I am taking into account the friends I know who are officials in mental hospitals without mental training.) As a mental nurse with long experience both in institute and private, I have no hesitation in saying that the worst patients I have ever handled have been the mismanaged cases of the so-called trained nurse. I do not say this with any desire to disparage my trained sisters; on the contrary, I have a strong admiration for nursing in all its branches, but I think for a patient suffering from this distressful malady there is nobody more fitted than the nurse who has given three years of her life to the study of mental work in all its phases.

I should just like to add a word for the institute I trained at—one of Scotland's private asylums. Every nurse worked hard and loyally for the certificate, and was encouraged in this by the kindness and hard-working officials.

The medical superintendent did more for suffering humanity and the betterment of conditions than the world knows. He held office for thirty years, during which time there never was mechanical restraint used. Bitter callousness on the part of the staff would never have been tolerated. As to healthful exercise, this was prescribed by those doctors in authority, and not by the staff. I am only one of crowds of nurses who would corroborate this were I to disclose the name of the institute.

"Matron" might be better employed looking around for a more educated type of mental probationer than content to sit in judgment upon the probationer she herself engages.—Yours faithfully,

